# Rethinking the Prognostic Role of Necrosis in Soft-Tissue Sarcoma: Multidisciplinary Insights from the Sarcoma Academy

**DOI:** 10.3390/cancers17111779

**Published:** 2025-05-26

**Authors:** Beata Bode-Lesniewska, Hans Roland Dürr, Dian Wang, Arash Naghavi, Julien Montreuil, Tim Fischer, Michelle Ghert, Alexander Lazarides, Lars Lindner, Javier Martin-Broto, Malena Mazza, Roberto Scanferla, Gabriela Studer, H. Tom Temple, Jay Wunder, Bruno Fuchs

**Affiliations:** 1 Institute for Pathology, LUKS University Hospital, Spitalstrasse 1, 6000 Luzern, Switzerland; 2 Schwerpunkt Tumororthopädie, Orthopädische Klinik der LMU München, Marchionistrasse 15, 81377 Munich, Germany; 3 Department of Radiation Oncology, Rush University Radiation Medicine, 1520, W. Harrison St., Chicago, IL 60607, USA; 4 Department of Radiation Oncology, Moffitt Cancer Center, 12902 USFF Magnolia Drive, Tampa, FL 33612, USA; 5 Department of Orthopaedics, Miller School of Medicine, University of Miami, 1600 NW 10th Ave 1140, Miami, FL 33136, USA; 6 Division of Musculoskeletal Radiology, Department of Radiology, KSW Sarcoma Center, Cantnal Hospital, 8401 Winterthur, Switzerland; 7 Department of Orthopaedics, University of Maryland, 110 S. Paca Street, College Park, MD 20742, USA; 8 Baltimore and Department of Surgery, McMaster University, Hamilton, ON L8S 4L8, Canada; 9 Department of Sarcoma, Moffitt Cancer Center, 12902 USF Magnolia Drive, Tampa, FL 33612, USA; 10 Onkologie, Medizinische Klinik und Poliklinik der LMU München, Marchionistrasse 15, 81377 Munich, Germany; 11 Medical Oncology Department, Fundación Jimenez Diaz University Hospital, 28040 Madrid, Spain; 12 University Hospital General de Villalba, 28400 Madrid, Spain; 13 Instituto de Investigacion Sanitaria Fundacion Jimenez Diaz (IIS-FJD), 28015 Madrid, Spain; 14 Pathology Department, Hospital Italiano de Buenos Aires, Buenos Aires C1199, Argentina; 15 Department of Orthopaedic Oncology and Reconstructive Surgery, Careggi University Hospital, 50139 Florence, Italy; 16 Department of Radiation Oncology, LUKS Sarcoma-IPU & Swiss Sarcoma Network, LUKS University Hospital, Spitalstrasse 1, 6000 Luzern, Switzerland; 17 Musculoskeletal Oncology Unit, Mount Sinai Hospital, University of Toronto, Toronto, ON M5S 1A1, Canada; 18 Department of Surgical Oncology, Princess Margaret Cancer Center, Toronto, ON M5G 2M9, Canada; 19 Division of Orthopaedic Surgery, Department of Surgery, University of Toronto, Toronto, ON M5S 1A1, Canada; 20 Department of Orthopedics & Orthopedic Oncology, LUKS Sarcoma-IPU & Swiss Sarcoma Network, LUKS University Hospital, Spitalstrasse 1, 6000 Luzern, Switzerland

**Keywords:** soft-tissue sarcoma, tumor necrosis, prognostic marker, neoadjuvant therapy, radiomics, real-world evidence, hybrid trial design, personalized medicine

## Abstract

**Background:** Soft-tissue sarcomas (STSs) represent a heterogeneous group of malignancies with widely varying treatment responses and biological behaviors. While spontaneous necrosis (present at diagnosis) is recognized in established sarcoma grading systems, the prognostic significance of therapy-induced necrosis remains uncertain. Inconsistent definitions, methodological variability, and clinical confounders further complicate the interpretation of necrosis as an independent prognostic marker. **Methods:** This communication synthesizes findings from an international, multidisciplinary webinar hosted by the Sarcoma Academy, critically assessing the utility of therapy-induced necrosis in STS management. Discussions encompassed surgical, pathological, oncological, and radiological perspectives, emphasizing how necrosis is defined, measured, and contextualized in patient care. **Results:** Heterogeneity in STS subtypes, varied treatment protocols, and sampling inconsistencies challenge the prognostic value of post-treatment necrosis. While substantial necrosis may sometimes signal effective therapy, it can also reflect the tumor’s aggressive nature. The panel underscored the utility of measuring the percentage of viable tumor cells, rather than necrosis alone, to obtain a more standardized and reproducible measure of therapy response. Emerging approaches—such as radiomics, molecular profiling, immune-based analyses, and real-world evidence (RWE) protocols—offer promising avenues for refining prognostication and guiding personalized therapy in STS. **Conclusions:** A focus solely on therapy-induced necrosis is insufficient to predict outcomes in STS. Instead, a multidisciplinary framework—combining standardized pathology protocols, quantification of viable tumor cells, advanced imaging, and innovative clinical trial designs—can better capture both treatment effects and underlying tumor biology. Future collaborative studies and hybrid trial methodologies are needed to determine which STS subgroups gain the most from intensified treatments aimed at maximizing necrosis, and how to balance such interventions with surgical considerations, toxicity, and overall patient well-being.

## Simple Summary

Soft-tissue sarcomas are rare cancers that can arise almost anywhere in the body and come in many different forms. Because they vary so much, doctors have a hard time predicting how each tumor will respond to treatment. One common way to judge success has been to look at how much of the tumor appears dead (called “necrosis”) after chemotherapy or radiotherapy. But this can be misleading, because some sarcomas are partly dead even before treatment, while others die off for reasons that have nothing to do with therapy. An international group of pathologists, surgeons, oncologists, and radiologists reviewed the latest evidence and agreed that a better yardstick is how much living (viable) tumor is left rather than how much is dead. They also highlighted new imaging techniques and shared pathology standards and real-world data projects that could make these measurements more precise and useful for patients.

## 1. Introduction

Soft-tissue sarcomas (STSs) are a heterogeneous group of malignancies arising from mesenchymal tissues, encompassing more than 50 distinct histological subtypes [1]. Despite their rarity, STSs collectively pose significant clinical challenges due to variable responses to treatment, a broad range of biological behaviors, and limited consensus on optimal management strategies. One key histopathological feature that has garnered attention in both research and clinical practice is tumor necrosis. Traditionally, the presence and extent of necrosis have been recognized as important parameters within established sarcoma grading systems, such as the French Federation of Cancer Centers Sarcoma Group (FNCLCC) system, which considers spontaneous necrosis (at diagnosis) alongside tumor differentiation and mitotic count to determine tumor grade and thereby stratify patient prognosis [2,3].

In high-grade osteosarcoma (and similarly in Ewing sarcoma), therapy-induced necrosis after neoadjuvant chemotherapy frequently correlates with improved overall and event-free survival, making it a statistically reliable—but not infallible—predictor [4,5,6]. Even in these bone sarcomas, some patients with high post-chemotherapy necrosis still experience relapses, emphasizing that necrosis alone does not capture the full complexity of tumor genetics, host factors, and microenvironment. Attempts to translate this necrosis-based concept to STS have proven more challenging. Unlike osteosarcoma—where relatively uniform histology and standardized chemotherapy protocols prevail—STSs include numerous subtypes with diverse biology and varied treatment regimens [7]. As a result, a simple link between extensive therapy-associated necrosis and better outcomes is not consistently observed in STSs. Several additional factors, such as distinguishing spontaneous from treatment-related necrosis, contribute to the difficulty of establishing necrosis as a universal prognostic marker [8,9,10].

Consequently, many centers now quantify the percentage of residual viable tumor cells (%VTC) as a more reproducible indicator of treatment effect [11,12].

In this paper, the term “necrosis” will be used in two principal contexts. Spontaneous necrosis refers to tumor cell death arising from inherent tumor biology (for example, outgrowing its blood supply), whereas preoperative therapy-induced necrosis indicates cell death attributable to neoadjuvant interventions—whether chemotherapy, radiotherapy, or combined modalities. Where relevant, “response” follows the concept outlined by Wang and Palm, highlighting the proportion of viable tumor cells remaining as a standardized index of therapeutic effect [11]. This approach aligns with a growing consensus that STS research and clinical practice require more reproducible, refined outcome measures.

Against this backdrop, the Sarcoma Academy (www.sarcoma.academy) (accessed on 24 May 2025) organized a webinar (https://www.youtube.com/watch?v=hJgkeReNpEQ accessed on 24 May 2025) on 12 December 2024 to examine the evolving role of post-treatment necrosis in STS. The panel comprised pathologists, surgeons, radiation oncologists, medical oncologists, and researchers, all exploring necrosis from multiple angles: pathological assessment; correlations with treatment outcomes; integration into established grading systems; and the application of novel diagnostic tools, such as molecular profiling, advanced imaging, and immunologic characterization. Panelists were deliberately drawn from leading institutions across North America, Europe, South America, and Asia, selected on the basis of recent peer-reviewed work on therapy-induced necrosis, to ensure a balanced, globally representative perspective. Special focus was placed on the role of % viable tumor cells and other comprehensive measures of response, aiming to bolster prognostic accuracy and standardize assessments across institutions. Through these multidisciplinary presentations and discussions, participants pinpointed key knowledge gaps, debated contradictory findings, and charted potential paths for collaborative research. In this paper, we synthesize those insights, critically review the current evidence on necrosis in STSs, and propose strategies to improve both clinical decision-making and future investigative directions.

## 2. Pathology Overview

### 2.1. Defining Necrosis in STS 

Necrosis in soft-tissue sarcomas can manifest through various microscopic patterns, each reflecting different underlying processes within the tumor. Coagulative necrosis is the most common type, featuring preservation of cellular outlines but loss of nuclear staining. Over time, these regions may display fibrosis of varying extent, hemorrhage residues, “ghost cells”, or pseudocystic change, complicating the task of determining whether the cell death is therapy-induced or spontaneous. For instance, in myxoid liposarcoma, a notable reduction in tumor cell density (often replaced by fibrous tissue) may follow radiotherapy, whereas in other subtypes, partially destroyed cellular components may represent a transitional stage rather than definitive necrosis. Importantly, spontaneous necrosis (arising from intrinsic tumor aggressiveness or poor vascularization) must be distinguished from therapy-induced necrosis, which can reflect neoadjuvant treatment efficacy [2,3,13]. For research protocols, ultrasound-guided core-needle biopsy—targeted with color-Doppler or intravenous contrast—can precisely sample macroscopic necrotic zones and provide quantitative tissue confirmation of imaging findings.

Given these complexities, many investigators have proposed quantifying the percentage of viable tumor cells (% viable cells) rather than necrosis alone [11,12,14]. Unlike necrosis, which can be challenging to measure consistently, “% viable cells” encompasses all non-viable tissue changes (necrosis, fibrosis, hemorrhage, inflammation, hyalinosis, or tissue maturation), providing a more holistic view of therapy impact. One caveat is that this snapshot fails to incorporate *baseline* necrosis—tumors already harboring extensive necrotic regions before therapy might still appear largely non-viable afterward, even without a strong cytotoxic effect. As a result, imaging-based or so-called “delta” approaches that compare pre- and post-treatment necrosis may capture treatment effectiveness more accurately. Nevertheless, “% viable cells” remains a more reproducible and standardized metric than conventional estimates of necrosis, aligning with emerging evidence on its prognostic value in STS [11,12].

Despite these advances, the FNCLCC grading system is designed exclusively for untreated tumors, thus focusing on spontaneous necrosis at initial diagnosis and excluding post-treatment changes. Moreover, pathology protocols for STSs—unlike standardized frameworks used in bone sarcomas—can vary in regard to how tissue blocks are selected, embedded, and interpreted [2,3,14,15,16]. Consensus-based guidelines for % viable-cell assessment are outlined in Section 4.3.

### 2.2. Heterogeneity of Necrosis

Multiple interrelated factors contribute to the broad variability in STS necrosis. Intrinsic tumor biology (growth kinetics, vascularization patterns, and molecular drivers) influences the degree of spontaneous necrosis and how the tumor responds to treatment. Treatment protocols also vary widely: standard fractionation radiotherapy may induce more gradual cell death than (ultra-)hypofractionated or dose-escalated regimens [17], and the addition of systemic chemotherapy can accelerate necrosis while simultaneously producing complex microscopic findings (e.g., inflammatory infiltrates or fibrotic transformation) [18].

The timing of surgery after neoadjuvant therapy further complicates interpretation. Tumors resected soon after high-dose radiotherapy or chemotherapy may not show the full extent of delayed cell death or tissue reorganization [19]. By contrast, an extended interval between therapy completion and resection can permit necrosis to evolve into fibrous or cystic areas that pathologists might interpret differently. In osteosarcoma or Ewing sarcoma, standardized grading systems (e.g., Huvos and Salzer-Kuntschik) explicitly quantify chemotherapy-induced necrosis, but no widely accepted equivalent exists for STSs beyond the EORTC-STBSG guidelines [14]. Developing or adapting such assessment schemas must account for STS heterogeneity to ensure that the measurement of necrosis is clinically meaningful.

This temporal dimension is therefore especially relevant to preoperative therapy-induced necrosis: the interval between completing neoadjuvant treatment and surgical resection can either mask or exaggerate the true amount of cell death related to preoperative treatment. Consequently, the adoption of “% viable cells” as a primary endpoint, alongside robust imaging assessments, remains key to harmonizing how STS therapy-induced response is measured across different institutions and timelines [11,12]. To facilitate inter-institutional consistency, we outline a template protocol—minimum one tissue block per cm of tumor, EORTC-STBSG-based viable-cell scoring, and concordant MRI/PET correlation—that will be circulated for multicenter validation and refinement.

## 3. Critical Analysis of Existing Evidence

### 3.1. Summaries of the Four Presented Studies

Dr. Dürr opened the webinar by sharing results from a large retrospective cohort of ~800 patients with both soft-tissue and bone sarcomas. This analysis compared spontaneous (pre-therapy) necrosis in resected, untreated tumors with post-neoadjuvant necrosis (from chemotherapy and/or radiotherapy) across multiple STS subtypes. Although necrosis levels were higher after neoadjuvant therapy, no clear improvement in overall survival was observed—contrasting with high-grade osteosarcoma, in which therapy-induced necrosis often predicts favorable outcomes. Notably, spontaneous necrosis at diagnosis often correlated with more aggressive tumor biology and poorer prognosis, underscoring its established role in the FNCLCC grading system. Dr. Dürr concluded that necrosis, whether spontaneous or therapy-induced, should be interpreted within a broader clinical context that includes tumor subtype, surgical margins, and timing of resection [20]. This viewpoint challenges the assumption—extrapolated from bone sarcomas—that a higher percentage of necrosis alone translates to improved survival in STS patients. He proposed % viable tumor cells as a potentially more informative metric [11,12].

#### 3.1.1. Bridging Perspective

Whereas Dr. Dürr’s large-cohort data cast doubt on the universal prognostic value of therapy-induced necrosis in heterogeneous STS, Dr. Wang’s work (presented next) offered a complementary perspective drawn from multicenter clinical trials [11,12]. The juxtaposition of these findings highlights the complexity of interpreting necrosis across different study designs, patient subsets, and therapeutic protocols.

#### 3.1.2. Dian Wang, Rush University—RTOG 9415 and 0630 Trials [11,21]

Dr. Wang presented findings from two Radiation Therapy Oncology Group (RTOG) trials evaluating neoadjuvant therapy in STS: RTOG 9415, which combined chemotherapy and radiation, and RTOG 0630, which used preoperative radiation alone (50 Gy in 25 fractions) [11,21]. Both trials defined a complete response as no viable tumor cells in the resected specimen, encompassing all forms of non-viable tissue (necrosis, fibrosis, and hemorrhage). Although such profound responses were uncommon, patients who achieved them had notably improved disease-free, metastasis-free, and overall survival. Dr. Wang attributed these outcomes to the intensity and consistency of neoadjuvant regimens and emphasized that near-complete or complete tumor obliteration—if attainable—may have substantial prognostic implications in STS. At the same time, he acknowledged practical limitations, such as subtype variability and real-world adherence issues.

#### 3.1.3. Arash Naghavi, Moffitt Cancer Center, HEAT Trial, and Precision Radiation

Dr. Naghavi discussed patients treated with radiation therapy alone, aiming for a “favorable pathologic response”, defined as ≥95% necrosis—again, consistent with minimal residual viable tumor cells [11,12,22]. Achieving such a high response fraction correlated with an increased likelihood of R0 resection and prolonged progression-free survival, suggesting that radiation treatment alone can independently induce meaningful therapy-related necrosis. He also introduced the HEAT trial’s “habitat-based” dose-escalation protocol, wherein hypoxic or resistant tumor regions (“radiomic habitats”) receive higher doses. Preliminary data indicate that this approach can increase the extent of non-viable tumor tissue, potentially enhancing local control. Complete (100%) response remains challenging, but even partial improvements in non-viable tissue can confer prognostic benefits.

#### 3.1.4. Julien Montreuil, University of Miami—Diverse Cohort Analysis

Dr. Montreuil presented an STS cohort treated with varied neoadjuvant regimens (including adjuvant brachytherapy) or none at all. Nearly half of the patients who received no neoadjuvant therapy exhibited ≥90% necrosis in their tumors, suggesting that spontaneous necrosis alone can be substantial. This finding challenges the notion that extensive necrosis automatically reflects effective treatment. Instead, Montreuil emphasized the role of surgical factors—particularly achieving clear margins and avoiding unplanned excisions—in predicting local control and overall survival, as confirmed later in the discussion. His group concluded that while therapy-induced necrosis remains an important observation, quantifying its actual contribution to the prediction of oncologic outcomes requires novel methodologies such as liquid biopsy or radiomics to isolate the specific “delta” necrosis attributable to treatment.

### 3.2. Challenges in Prognostication

Accurately interpreting necrosis in STSs is hampered by inconsistent thresholds—some protocols consider ≥90% necrosis as a good response, whereas others require ≥95% or even complete necrosis to define a robust response. This variability impedes direct comparisons across studies and can yield conflicting clinical recommendations. Furthermore, pathology sampling methods differ significantly: some centers embed the entire specimen, while others sample only selected tumor blocks, risking under- or overestimation of necrosis. Multiple confounders also obscure straightforward survival correlations. Treatment approaches (single-agent vs. multi-agent chemotherapy, and standard- vs. (ultra-)hypo-fractionated radiotherapy), tumor grade, histological subtype, and margin status each exert strong influences on outcomes, potentially overshadowing necrosis as a prognostic indicator. High-risk STSs, for instance, which are usually also high-grade tumors, may already harbor extensive spontaneous necrosis, complicating efforts to distinguish additional therapy-induced necrosis. This is analogous to the RECIST response, which is neither consistently predictive nor sufficient on its own [23]. Looking forward, radiomics could potentially help to address this by quantifying changes between baseline and post-therapy imaging, thereby enabling a more precise evaluation of the impact of neoadjuvant treatment on tumor viability [24,25].

### 3.3. Insights and Integrations

Despite these complexities, robust evidence shows that intensive neoadjuvant regimens can induce notable necrosis in STSs. However, local tumor cell death does not always translate into better systemic control: high-grade subtypes can appear sensitive initially but still relapse or harbor undetected micrometastases. Conversely, data from large cooperative trials (e.g., RTOG 9415, 0630), as well as retrospective analyses, reveal that near-complete or complete pathologic response correlates with improved disease-free and metastasis-free survival in certain patient cohorts [26].

Such heterogeneous results underline the need to integrate necrosis measurements with other markers—molecular or immunologic profiles, surgical margin quality, and advanced imaging—to assemble a more personalized prognostic framework. In this scenario, therapy-induced necrosis becomes one key piece among many, rather than a standalone predictor of patient outcomes. By situating necrosis within a multifactorial matrix, clinicians and researchers can better account for unique tumor features, local therapy variables, and the risk of occult metastatic spread, ultimately guiding more tailored treatment strategies.

## 4. Consensus Statements

### 4.1. Therapy-Induced Necrosis as a Standalone Marker

Although chemotherapy-induced necrosis is well established and clinically relevant for bone sarcomas, it remains a controversial predictor in STSs treated with preoperative radio- or chemotherapy when viewed in isolation. This ambiguity supports transitioning toward more precise metrics, such as % viable tumor cells, which can standardize and reproducibly assess therapy response. Moreover, rather than focusing solely on necrosis, a broader concept of tissue changes, including necrosis, fibrosis, edema, and structural remodeling, may be captured via MRI and potentially correlate with outcomes in localized STSs.

### 4.2. Incorporating Other Factors

A more reliable approach embeds necrosis data into a multifactorial framework that also takes into account surgical margins, histological subtype, immunologic features, and advanced imaging. Such an integrated strategy acknowledges that surgical margins often dominate the evaluation of local control and survival outcomes, regardless of necrosis levels. Additionally, using % viable cells as a primary endpoint can unify the quantification of treatment effects across diverse STS subtypes.

### 4.3. Standardization and Protocols

A unifying theme from the webinar was the urgent need for standardized pathological assessment and consensus-based guidelines for measuring and reporting necrosis. Because the FNCLCC system covers only spontaneous necrosis, recent work by Wardelmann et al. [14] highlights the urgent need for a consensus protocol that combines pathological and imaging criteria for therapy-induced necrosis. Harmonizing criteria for differentiating viable tumor tissue from necrotic regions—whether therapy-induced or spontaneous—would enhance data comparability among institutions. This standardization is vital for enabling rigorous meta-analyses and promoting robust, collaborative research.

### 4.4. Future Research Directions (Innovative Methodologies)

Because of STS complexity, innovative methodologies can surpass basic necrosis thresholds in prognostication. Approaches such as radiomics-based MRI, digital pathology, molecular biomarkers, and immune profiling may deepen our understanding of tumor behavior. Meanwhile, real-world evidence (RWE) and pragmatic trials will allow for the inclusion of larger, more diverse populations, capturing real-life clinical environments and generating more applicable insights. Embedding patient-reported outcomes (PROs) and systematic toxicity monitoring will ensure that therapy intensification is balanced with patient well-being. In addition to MRI-based radiomic analyses, FDG-PET can quantify metabolic response in soft-tissue sarcomas, aiding in the distinction between viable tumor tissue and necrotic or fibrotic areas [27,28].

### 4.5. Target Trial Emulation and Hybrid Designs

Because of STS rarity and diversity, target trial emulation and other hybrid designs offer the chance to generate high-level evidence without the constraints of conventional randomized controlled trials (RCTs) [29,30]. These alternative methodologies enable researchers to leverage observational data and advanced statistical techniques, thereby creating RCT-like conditions in real-world settings. Hybrid RCTs combine elements of traditional randomized trials with real-world data collection or adaptive methods—such as broad inclusion criteria, embedded observational cohorts, or continuous data analysis—to reflect routine practice more closely. This blended model can yield nuanced assessments of necrosis as a prognostic or predictive factor in STS, while also capturing the complexities of everyday clinical management.

## 5. Discussion

### 5.1. Expert Opinions and Points of Agreement

A central takeaway from the Sarcoma Academy webinar was the intrinsic complexity of therapy-induced necrosis in the prognostic landscape of STS. While some participants observed strong correlations between extensive necrosis and favorable outcomes, others cautioned that high necrosis can also reflect an inherently aggressive tumor phenotype rather than a purely robust treatment response.

Standardized pathology protocols for evaluating necrosis and viable tumor cells emerged as a clear need. Studies linking necrosis to outcome [11,12] often include all non-viable components (e.g., necrosis, fibrosis, hemorrhage, and inflammation), leading many experts to suggest that percent viable tumor cells might serve as a more uniform method for quantifying response across different institutions.

Furthermore, surgical margins—rather than necrosis alone—remained a consistent determinant of local control and overall survival. Even a seemingly necrotic tumor may recur if resection is incomplete. Meanwhile, the presence of residual viable cells after intensive therapy underscores the resilience of certain STS subtypes. This observation reinforces the importance of high-quality surgery and underscores the value of novel biomarkers or imaging strategies that go beyond necrosis alone to predict treatment impact accurately.

It is also crucial to distinguish quality of clinical management (e.g., suboptimal staging and unplanned excisions) from the tumor’s intrinsic response to therapy. While poor management can negatively affect outcomes, it does not necessarily reflect the tumor’s innate sensitivity or resistance. Rather, these factors highlight the importance of specialized, multidisciplinary centers that can integrate accurate diagnostics with the most effective treatment strategies.

### 5.2. Clinical and Research Implications

#### 5.2.1. Clinical Context

Attempts to escalate therapy solely to increase necrosis (e.g., adding more chemotherapy agents, higher radiation doses, or hyperthermia) remain controversial unless supported by robust data linking high necrosis to demonstrable survival benefits. Near-complete necrosis can be achieved in certain high-intensity or multimodal regimens, but results vary substantially across STS subtypes and clinical settings. As a result, individualized decision-making is essential—patient comorbidities, tumor subtype biology, sequencing of therapy, and the likelihood of achieving negative margins must all be weighed when considering treatment intensification. Because necrosis-response relationships differ by histotype, developing subtype-specific guidelines for interpreting viable-cell percentages is a recognized priority for forthcoming multicenter collaborations.

#### 5.2.2. Research Perspectives

From a research standpoint, real-world evidence (RWE) and pragmatic trials provide broader patient populations and more authentic practice patterns than conventional randomized trials. Capturing diverse therapeutic regimens, including standard or hypofractionated radiotherapy; combination chemotherapy; and specialized interventions, like hyperthermia, can elucidate how best to induce meaningful necrosis while minimizing toxicity. Embedding patient-reported outcomes (PROs) in these frameworks ensures that quality of life remains central to any strategy aimed at maximizing necrosis.

Adaptive and hybrid trial designs can further refine therapy intensification. Such methodologies enable investigators to adjust interventions based on interim analyses, thereby identifying the subset of patients most likely to benefit from enhanced necrosis. In the context of rare tumors like STSs, where classical RCTs are often impractical, these adaptive designs may uncover more nuanced correlations between therapy-induced necrosis and survival endpoints. Emerging analyses of long-term trial data also suggest that continuous, nuanced metrics (e.g., dimensional variation) outperform binary or categorical response criteria in predicting survival, although their clinical utility remains uncertain [23]. These consensus views are further underpinned by aggregated retrospective cohort data from multiple high-volume sarcoma centers and national registries, providing a real-world evidential base for our recommendations. In parallel, international consensus initiatives—linking groups such as EORTC-STBSG, NRG Oncology, and EURACAN—are drafting a unified pathology-plus-imaging response score, which forthcoming pragmatic trials will prospectively validate across diverse STS subtypes. Practically, we recommend that clinicians (i) report the percentage of viable tumor cells using a harmonized scoring sheet; (ii) correlate this with post-therapy MRI/PET findings obtained at the same time-point; and (iii) document margin status in the same report, forming a concise three-item checklist for treatment decisions.

#### 5.2.3. Radiologist’s Point of View in Necrosis Assessment

In assessing therapy-induced necrosis in soft-tissue sarcomas (STS), imaging plays a critical role in providing non-invasive insights into tumor response. Contrast-enhanced MRI is the primary imaging tool used, with necrosis typically defined as low signal intensity on T1-weighted imaging, high signal intensity on T2-weighted imaging, and absence of contrast enhancement, best evaluated with regard to the pre-treatment baseline examination. Quantitative imaging, such as diffusion-weighted imaging can be used for the evaluation of therapy response [31]. This imaging technique is well-established as a prognostic factor, as demonstrated by Crombé et al. (2019), who showed that MRI features correlate with histological grade and patient outcomes in STSs [7]. Additionally, PET/CT and PET/MRI have proven complementary to MRI by offering functional imaging that provides further insight into tumor metabolism and necrotic areas, as is crucial for monitoring therapeutic response. Beyond visual assessment, radiomics-based extraction of high-dimensional imaging features from serial MRI or PET datasets enables quantitative tracking of necrotic and viable tumor compartments over time, thereby providing an objective and reproducible biomarker of treatment response during follow-up [24,25]. Recent studies have also highlighted the utility of these imaging techniques in rare histotypes. For instance, Di Masi et al. demonstrated that MRI findings, including necrosis and tumor size, were associated with metastatic outcomes in Clear Cell Sarcoma, a rare STS subtype, reinforcing the relevance of imaging in therapeutic monitoring [32]. However, in certain high-grade and aggressive STS subtypes, such as Alveolar Soft Part Sarcoma (ASPS) and Malignant Solitary Fibrous Tumor (SFT), imaging may show limited or no visible necrosis, even in cases where the tumors are biologically aggressive. This poses a challenge for imaging-based necrosis assessment and highlights the need for careful consideration of imaging findings in these histotypes. As such, a comprehensive evaluation, combining imaging with other diagnostic modalities, is crucial for accurately assessing the therapeutic response in these tumors.

### 5.3. Considering the Consequences of Escalating Preoperative Therapy to Increase Necrosis

A pivotal question raised during the webinar concerned whether clinicians should intensify or prolong neoadjuvant therapy (e.g., additional chemotherapy, hyperthermia, and higher radiotherapy doses) specifically to push necrosis beyond certain thresholds. One major concern is that longer or more aggressive regimens may inadvertently promote resistant clones, potentially offsetting local gains by heightening the risk of micrometastatic spread. Such regimens can also delay surgery, allowing new tumor cells more time to disseminate.

Because STS subtypes vary greatly in their biology and therapy sensitivities, universal escalation of preoperative therapy may be a strategy that does not benefit every patient. Indeed, surgical margin status, inherent tumor aggressiveness (e.g., grade), and the host’s immunologic milieu can overshadow raw necrosis percentages in shaping outcomes. Consequently, while near-complete necrosis sometimes correlates with better local and/or disease-free survival, its value as an independent prognostic marker must be balanced against the risk of selecting resistant clones and postponing definitive surgical resection.

Moving forward, research should clarify which STS subtypes or clinical scenarios stand to gain the most from extended neoadjuvant regimens, and which tumors may be better addressed by earlier surgery followed by adjuvant therapy. Until these data emerge, a measured, individualized approach remains prudent—assessing whether increased local tumor-cell kill outweighs the potential for systemic compromise and surgical delays.

**Figure 1:**
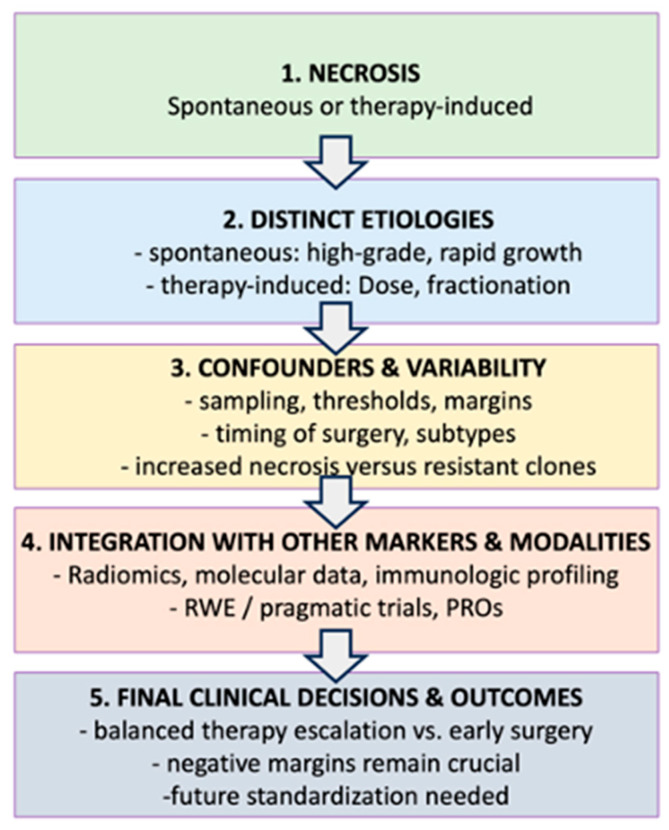
Flow Chart Summarizing Necrosis in Soft Tissue Sarcomas: From Etiologies to Clinical Decision-Making.

Figure 1 and Table 1, together, provide an integrated overview of necrosis in STSs. The flowchart on the left outlines the logical pathway from recognizing necrosis (whether spontaneous or preoperative therapy-induced) to understanding how various confounders—such as sampling bias, threshold inconsistencies, and margin status—may influence its interpretation. Each node in the flowchart is labeled (A–E) to match the corresponding entries in Table 1. The table expands on these labels with concise bullet points, comparing spontaneous necrosis to therapy-induced necrosis, detailing key factors (e.g., timing of surgery, tumor biology, dose fractionation, and immune context), and highlighting clinical implications. By consulting both visuals side by side, readers can quickly grasp the broader conceptual sequence shown in the flowchart while referencing the table for more granular or comparative details relevant to clinical decision-making and future research directions.

## 6. Conclusions

Soft-tissue sarcomas (STSs) represent a diverse spectrum of malignancies whose management often requires nuanced, multidisciplinary approaches. Although tumor necrosis is an established histopathological feature in bone sarcomas, its role as a definitive and independent prognostic indicator in STS remains far less certain. Presentations at the Sarcoma Academy webinar highlighted how necrosis may be shaped by both intrinsic tumor biology and therapeutic interventions—chemotherapy, radiotherapy, or combined modalities—leading to inconclusive or confounded associations with long-term outcomes.

**1.**  **Therapy-induced necrosis alone is not a definitive prognostic indicator in STS.**
  High levels of necrosis may reflect either inherent tumor aggressiveness or effective therapy, complicating interpretation—especially when differentiating spontaneous necrosis (as used in the FNCLCC system) from treatment-related cell death. A uniform pathologic protocol that measures % viable tumor cells may provide an alternative or complimentary approach, but consistency is critical. However like necrosis, the potential prognostic value of % viable tumor cells is also likely to depend on factors such as tumor subtype, timing of resection, and surgical margins.
**2.**  **Percent of viable tumor cells (rather than necrosis alone) offers a more reliable measure of therapeutic effect.**
  Reporting the percentage of viable tumor cells (%VTC) provides a uniform endpoint—the amount of living tissue that remains post-therapy. All other treatment-related changes (necrosis, fibrosis, hemorrhage, and inflammation) are classified as non-viable, a simplification that reduces inter-observer variation and enables more reliable cross-study comparison.
**3.**  **Complete pathologic response is rare but may correlate with better outcomes in selected settings.**
  In certain prospective trials (e.g., RTOG 9514, 0630), 19–27% of patients achieved complete absence of viable tumor cells, often linked to improved survival (and retrospective series [26]). However, due to STS heterogeneity, potential toxicities, and the possibility of selecting resistant clones by delaying surgery, universal escalation of therapy to maximize necrosis remains neither routinely advisable nor consistently beneficial.
**4.**  **Surgical resection margins trump necrosis and subtype for local control.**
  Securing negative surgical margins remains the cornerstone of local control in STS and outweighs both necrosis extent and histological subtype. Although tumor biology and other factors contribute, margin status consistently exerts the strongest influence on recurrence risk.
**5.**  **Pathologic and radiologic measures of response are complementary but distinct.**
  Pathologists assess histological changes, including necrosis, viable cells, and fibrosis, whereas radiologists evaluate imaging-based criteria (e.g., tumor shrinkage and metabolic shifts). Integrating both perspectives provides a more comprehensive picture of therapeutic impact.
**6.**  **Jointly agreed pathology-plus-imaging protocols are essential for comparability across centers.**
  Inconsistent thresholds and variable pathological sampling underscore the need for universally accepted protocols (for example, [13]). Harmonizing radiological response criteria (e.g., RECIST or PERCIST) with standardized pathology procedures is similarly essential.
**7.**  **Integrating imaging-based biomarkers, molecular data, and immunologic profiling may enhance prognostic accuracy.**
  Techniques such as radiomics, genomic analysis, and immune microenvironment characterization can provide deeper insights into tumor behavior, enabling more precise risk stratification.
**8.**  **Real-world evidence and pragmatic trials can circumvent some limitations of traditional RCTs, improving clinical relevance.**
  Broader, more inclusive study designs in RWE frameworks capture a wider range of patient and treatment variations, refining our understanding of necrosis, % viable cells, and response in everyday STS care.
**9.**  **Patient-reported outcomes and toxicity data should be standard secondary endpoints, ensuring treatments remain patient-focused.**
  Balancing efficacy with side-effect profiles is imperative, ensuring any push to increase necrosis remains aligned with patient well-being and quality of life.
**10.**  **Target trial emulation and hybrid study designs can yield better causal insights in rare, heterogeneous STSs.**
  These methodologies enable more nuanced exploration of necrosis and % viable cells in smaller or diverse patient populations, approximating randomized conditions while reflecting real-world clinical practice.

Overall, therapy-induced necrosis is but one facet of STS prognostication. Although near-complete response appears promising—particularly in combined chemo-radiation regimens—cohesive research efforts and uniform assessment protocols remain vital to unlocking necrosis’s true clinical significance. By embedding necrosis evaluation into a broader, patient-centered care model—one that considers margins, molecular profiles, and overall functional outcomes—treatment strategies can be more precisely tailored to maximize benefit while minimizing risk.

## Figures and Tables

**Table 1:** Table Summarizing Necrosis in Soft Tissue Sarcomas: From Etiologies to Clinical Decision-Making

Label	Topic or Factor	Detail/Key Points	Connection to Flow Chart
A	Spontaneous Necrosis	- usually indicates inherent tumor aggressiveness, poor vascularization. - not necessarily reflective of treatment efficacy. - correlates with worse outcomes in all STS subtypes.	Referenced at node (2) for difference in etiologies.
B	Preoperative Therapy induced Necrosis	- driven by chemo, radiation (or combination). - percent necrosis often used as a proxy for treatment response. - high necrosis (^90–95%) can correlate with better DFS/MFS in specific subgroups.	Node (2) to compare with spontaneous necrosis.
C	Confounders & Variability	- sampling bias, threshold inconsistency (10%, 90%, etc.). - timing of surgery vs. therapy (risk of resistant clones). - overlapping effect of surgical margins.	Node (3) describes multiple confounders that Influence necrosis interpretation.
D	Integration with other markers/Modalities	- radiomics, molecular profiling, immunologic infiltration data. - real-world evidence (RWE), pragmatic trials, & patient reported outcomes (PROs). - guides personalizing therapy beyond necrosis alone.	Node (4) where necrosis merges with advanced diagnostics ad trial designs.
E	Clinical Decisions/Future Directions	- balancing therapy escalation vs. risk of overtreatment/toxicity. - importance of early resection, negative margins. - standardized protocols for measuring necrosis in prospective studies.	Node (5) final step in the flowchart: linking necrosis to actual treatment outcomes.

Table 1 Provide an integrated overview of necrosis in STS. The table expands on these labels with concise bullet points, comparing spontaneous necrosis to therapy-induced necrosis, detailing key factors (e.g., timing of surgery, tumor biology, dose fractionation, and immune context), and highlighting clinical implications. By consulting both visuals side by side, readers can quickly grasp the broader conceptual sequence shown in the flow chart while referencing the table for more granular or comparative details relevant to clinical decision-making and future research directions.

## Data Availability

No new data were created or analyzed in this study. The original contributions presented in this study are included in the article. Further inquiries can be directed to the corresponding author(s).

## References

[1] Antonescu C., Blay J. (2020). WHO Classification of Tumours: Soft Tissue and Bone Tumours.

[2] Gannon N.P., Stemm M.H., King D.M., Bedi M. (2019). Pathologic necrosis following neoadjuvant radiotherapy or chemoradiotherapy is prognostic of poor survival in soft tissue sarcoma. J. Cancer Res. Clin. Oncol..

[3] Salah S., Lewin J., Amir E., Abdul Razak A. (2018). Tumor necrosis and clinical outcomes following neoadjuvant therapy in soft tissue sarcoma: A systematic review and meta-analysis. Cancer Treat. Rev..

[4] Patel N., Werenski J.O., Gonzalez M.R., Clunk M.J., McCadden M.R., Richard A., Chebib I., Hung Y.P., Nielsen G.P., Lozano-Calderon S.A. (2024). Tumor Necrosis Drives Prognosis in Osteosarcoma: No Difference in Chemotherapy Response and Survival Between Chondroblastic and Osteoblastic Osteosarcoma. Surg. Oncol..

[5] Lozano-Calderon S.A., Albergo J.I., Groot O.Q., Merchan N.A., El Abiad J.M., Salinas V., Gomez Mier L.C., Montoya C.S., Ferrone M.L., Ready J.E. (2023). Complete tumor necrosis after neoadjuvant chemotherapy defines good responders in patients with Ewing sarcoma. Cancer.

[6] Wunder J.S., Paulian G., Huvos A.G., Heller G., Meyers P.A., Healey J.H. (1998). The histological response to chemotherapy as a predictor of the oncological outcome of operative treatment of Ewing sarcoma. J. Bone Jt. Surg. Am..

[7] Crombe A., Marcellin P.J., Buy X., Stoeckle E., Brouste V., Italiano A., Le Loarer F., Kind M. (2019). Soft-Tissue Sarcomas: Assessment of MRI Features Correlating with Histologic Grade and Patient Outcome. Radiology.

[8] Nystrom H., Jonsson M., Nilbert M., Carneiro A. (2023). Immune-cell infiltration in high-grade soft tissue sarcomas; prognostic implications of tumor-associated macrophages and B-cells. Acta Oncol..

[9] Qi L., Xu R., Ren X., Zhang W., Yang Z., Tu C., Li Z. (2022). Comprehensive Profiling Reveals Prognostic and Immunogenic Characteristics of Necroptosis in Soft Tissue Sarcomas. Front. Immunol..

[10] Vogin G., Lepage M., Salleron J., Cuenin M., Blum A., Gondim Teixeira P.A. (2024). Evaluation of the Prognostic Value of Pretherapeutic Magnetic Resonance Imaging in Predicting Soft Tissue Sarcoma Radiation Response: A Retrospective Study from a Large Institutional Sarcoma Imaging Database. Cancers.

[11] Wang D., Harris J., Kraybill W.G., Eisenberg B., Kirsch D.G., Ettinger D.S., Kane J.M., Barry P.N., Naghavi A., Freeman C.R. (2023). Pathologic Complete Response and Clinical Outcomes in Patients With Localized Soft Tissue Sarcoma Treated With Neoadjuvant Chemoradiotherapy or Radiotherapy: The NRG/RTOG 9514 and 0630 Nonrandomized Clinical Trials. JAMA Oncol..

[12] Boulouta A., Kyriazoglou A., Kotsantis I., Economopoulou P., Anastasiou M., Pantazopoulos A., Kyrkasiadou M., Moutafi M., Gavrielatou N., Zazas E. (2024). Pathologic complete response in patients with localized soft tissue sarcoma treated with neoadjuvant therapy and its correlation with clinical outcomes: A systematic review. Cancer Treat. Rev..

[13] Smolle M.A., Andreou D., Tunn P.U., Szkandera J., Liegl-Atzwanger B., Leithner A. (2017). Diagnosis and treatment of soft-tissue sarcomas of the extremities and trunk. EFORT Open Rev..

[14] Wardelmann E., Haas R.L., Bovee J.V., Terrier P., Lazar A., Messiou C., LePechoux C., Hartmann W., Collin F., Fisher C. (2016). Evaluation of response after neoadjuvant treatment in soft tissue sarcomas; the European Organization for Research and Treatment of Cancer-Soft Tissue and Bone Sarcoma Group (EORTC-STBSG) recommendations for pathological examination and reporting. Eur. J. Cancer.

[15] Sugita S., Tanaka K., Oda Y., Nojima T., Konishi N., Machida R., Kita R., Fukuda H., Ozaki T., Hasegawa T. (2024). Prognostic evaluation of the Ki-67 labeling system in histological grading of non-small round cell sarcoma: A supplementary analysis of a randomized controlled trial, JCOG1306. Jpn. J. Clin. Oncol..

[16] Stergioula A., Kormas T., Kokkali S., Memos N., Pantelis E., Pouloudi D., Agrogiannis G. (2024). What Is the Prognostic Value of the Pathologic Response after Neoadjuvant Radiotherapy in Soft Tissue Sarcoma? An Institutional Study Using the EORTC–STBSG Response Score. Cancers.

[17] Boxberg M., Langer R., Woertler K., Knebel C., Rechl H., von Eisenhart-Rothe R., Weichert W., Combs S.E., Hadjamu M., Roper B. (2022). Neoadjuvant Radiation in High-Grade Soft-Tissue Sarcomas: Histopathologic Features and Response Evaluation. Am. J. Surg. Pathol..

[18] Gennaro N., van der Loo I., Reijers S.J.M., van Boven H., Snaebjornsson P., Bekers E.M., Bodalal Z., Trebeschi S., Schrage Y.M., van der Graaf W.T.A. (2024). Heterogeneity in response to neoadjuvant radiotherapy between soft tissue sarcoma histotypes: Associations between radiology and pathology findings. Eur. Radiol..

[19] Hanafi H., Freeman C.R., Tsui J., Ramia P., Turcotte R., Aoude A., Bozzo A., Cury F.L. (2024). A retrospective study on the comparision of pathological tumour necrosis of conventional versus ultrahypofractionated preoperative radiotherapy in localised extremity soft tissue sarcoma and its correlation with clinical outcomes: A retrospective study on the comparision of pathological tumour necrosis of CONV-RT versus UHYPO-RT preoperative radiotherapy in localised extremity soft tissue sarcoma and its correlation with clinical outcomes. Pract. Radiat. Oncol..

[20] Fromm J., Klein A., Kirilova M., Lindner L.H., Nachbichler S., Holzapfel B.M., Goller S.S., Knosel T., Durr H.R. (2024). The Effect of chemo- and radiotherapy on tumor necrosis in soft tissue sarcoma- does it influence prognosis?. BMC Cancer.

[21] Wang D., Abrams R.A. (2014). Radiotherapy for soft tissue sarcoma: 50 years of change and improvement. Am. Soc. Clin. Oncol. Educ. Book.

[22] Naghavi A.O., Bryant J.M., Kim Y., Weygand J., Redler G., Sim A.J., Miller J., Coucoules K., Michael L.T., Gloria W.E. (2024). Habitat escalated adaptive therapy (HEAT): A phase 2 trial utilizing radiomic habitat-directed and genomic-adjusted radiation dose (GARD) optimization for high-grade soft tissue sarcoma. BMC Cancer.

[23] Vanzulli A., Vigorito R., Buonomenna C., Palmerini E., Quagliuolo V., Broto J.M., Lopez Pousa A., Grignani G., Brunello A., Blay J.Y. (2025). Redefining radiologic responses in high-risk soft-tissue sarcomas treated with neoadjuvant chemotherapy: Final results of ISG-STS 1001, a randomized clinical trial. ESMO Open.

[24] White L.M., Atinga A., Naraghi A.M., Lajkosz K., Wunder J.S., Ferguson P., Tsoi K., Griffin A., Haider M. (2023). T2-weighted MRI radiomics in high-grade intramedullary osteosarcoma: Predictive accuracy in assessing histologic response to chemotherapy, overall survival, and disease-free survival. Skelet. Radiol..

[25] Isaac C., Kavanagh J., Griffin A.M., Dickie C.I., Mohankumar R., Chung P.W., Catton C.N., Shultz D., Ferguson P.C., Wunder J.S. (2022). Radiological progression of extremity soft tissue sarcoma following pre-operative radiotherapy predicts for poor survival. Br. J. Radiol..

[26] Bonvalot S., Wunder J., Gronchi A., Broto J.M., Turcotte R., Rastrelli M., Papai Z., Radaelli S., Lindner L.H., Shumelinsky F. (2021). Complete pathological response to neoadjuvant treatment is associated with better survival outcomes in patients with soft tissue sarcoma: Results of a retrospective multicenter study. Eur. J. Surg. Oncol..

[27] Chodyla M., Demircioglu A., Schaarschmidt B.M., Bertram S., Morawitz J., Bauer S., Podleska L., Rischpler C., Forsting M., Herrmann K. (2021). Evaluation of the Predictive Potential of 18F-FDG PET and DWI Data Sets for Relevant Prognostic Parameters of Primary Soft-Tissue Sarcomas. Cancers.

[28] Crompton J.G., Armstrong W.R., Eckardt M.A., Seyedroudbari A., Tap W.D., Dry S.M., Abt E.R., Calais J., Herrmann K., Czernin J. (2022). (18)F-FLT PET/CT as a Prognostic Imaging Biomarker of Disease-Specific Survival in Patients with Primary Soft-Tissue Sarcoma. J. Nucl. Med..

[29] Hernan M.A., Wang W., Leaf D.E. (2022). Target Trial Emulation: A Framework for Causal Inference From Observational Data. JAMA.

[30] Hernan M.A., Robins J.M. (2016). Using Big Data to Emulate a Target Trial When a Randomized Trial Is Not Available. Am. J. Epidemiol..

[31] Subhawong T.K., Jacobs M.A., Fayad L.M. (2014). Insights into quantitative diffusion-weighted MRI for musculoskeletal tumor imaging. AJR Am. J. Roentgenol..

[32] Di Masi P., Colangeli M., Simonetti M., Bianchi G., Righi A., Bilancia G., Palmerini E., Crombe A., Spinnato P. (2025). Clear Cell Sarcoma of Soft Tissues: Radiological Analysis of 14 Patients-MRI Findings Related to Metastatic Disease. Diagnostics.

